# The role of gut microbes in production of aromatic carboxaldehydes

**DOI:** 10.1080/19490976.2026.2632979

**Published:** 2026-02-21

**Authors:** Manish Kumar, Rachel Son, Sarah M. Preston, Robert W. P. Glowacki, Kelley M. Carr, Jiyeon Kim, Jin Z. Ma, Philip P. Ahern, Jan Claesen, Naseer Sangwan, Florian Rieder, Ina Nemet

**Affiliations:** a Department of Cardiovascular & Metabolic Sciences, Cleveland Clinic Research, Cleveland, OH, USA; b Center for Microbiome & Human Health, Cleveland Clinic, Cleveland, OH, USA; c Department of Biology, Case Western Reserve University, Cleveland, OH, USA; d Biology Department, Hope College, Holland, MI, USA; e Department of Inflammation & Immunity, Cleveland Clinic Research, Cleveland, OH, USA; f Case Western Reserve University School of Medicine, Cleveland Clinic Lerner College of Medicine, Cleveland, OH, USA; g Department of Gastroenterology, Hepatology and Nutrition, Digestive Disease Institute, Cleveland Clinic, Cleveland, OH, USA; h Program for Global Translational Inflammatory Bowel Disease, Cleveland Clinic, Cleveland, OH, USA

**Keywords:** Gut microbes, aromatic amino acids, aromatic carboxaldehydes, benzaldehyde, 4-hydroxybenzaldehyde, 4-imidazolecarboxaldehyde, indole-3-carboxaldehyde, inflammatory bowel disease, IBD, Crohn's disease, ulcerative colitis

## Abstract

Gut microbes play an important role in maintaining our health through the alteration of available nutrients and by the production of small molecules/metabolites. Indole-3-carboxaldehyde (I3A) is an aromatic carboxaldehyde (ArA) synthesized by gut microbes from the aromatic amino acid tryptophan, and has been mechanistically linked to antitumor activity, intestinal homeostasis, and metabolic syndrome. However, the capacity of gut microbes to produce other ArAs and their associations with host health remains largely unexplored due to a lack of methods for their detection and quantification. A stable isotope dilution mass spectrometry method for quantifying ArAs from all four aromatic amino acids (benzaldehyde (BA), 4-hydroxybenzaldehyde (4HBA), and 4-imidazolecarboxaldehyde (4IA) in addition to I3A) based on derivatization with 3-methoxyphenylhydrazine was developed and validated. Fecal levels of I3A and 4HBA were reduced in both human and mouse feces after a cocktail of nonabsorbable antibiotics depleted the gut microbiota, while the same treatment reduced BA in humans and 4IA in mice. Further, we identified multiple commensals with the capacity to produce selected ArAs in culture and showed that individuals with Crohn's disease, but not in those with ulcerative colitis, have lower fecal levels of I3A and 4HBA, relative to non-inflammatory bowel disease controls.

## Graphical abstract

**Figure:**
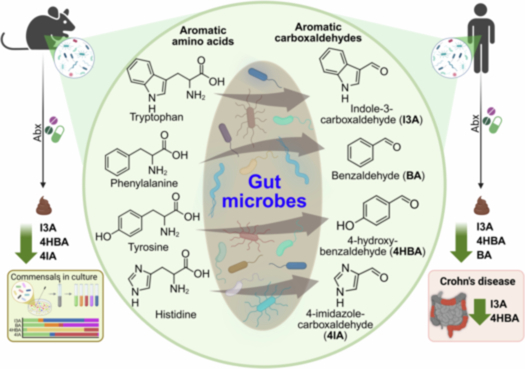

## Introduction

Gut microbes play an important role in maintaining our health with multiple human diseases being associated with altered gut-microbial structure and function.^1-3^ Gut microbes interact with the host through the alteration of available nutrients and production of small molecules. The microbial fermentation of dietary fibers into short chain fatty acids (SCFAs), for example, has been associated with improved host metabolism and enhancement of regulatory T-cells.^4^ Furthermore, as an important fuel for intestinal epithelial cells, SCFAs play an important role in maintaining the gut barrier.^5-8^ Secondary bile acids, produced by the microbial modifications of host derived bile acids, have a wide range of effects on host metabolic and immune activity^9^^,^^10^ and disturbances in bile acid homeostasis have been observed in individuals with inflammatory bowel disease (IBD).^11^ However, the metabolic potential of gut microbes is still largely unexplored.

Indole-3-carboxaldehyde (I3A) is an aromatic carboxaldehyde (ArA) produced by gut microbes from the amino acid tryptophan (Trp)^12^ and it is mechanistically linked to antitumor activity, intestinal homeostasis, and metabolic syndrome through engaging aryl hydrocarbon receptor (AhR).^12-16^ Furthermore, benzaldehyde (BA) and 4-hydroxybenzaldehyde (4HBA), aromatic carboxaldehydes derived from respective enzymatic degradation of phenylalanine (Phe) or tyrosine (Tyr), play a role in food flavoring and are often identified as possible biomarkers of certain diets and some health disorders.^17-24^ However, capacity of gut microbes to produce carboxaldehydes from these two aromatic amino acids as well as histidine (His) remains unexplored.

Here, we developed and validated a quantitative stable isotope dilution liquid chromatography mass spectrometry (LC-MS/MS) method for quantifying ArAs based on derivatization with 3-methoxyphenylhydrazine (3MPH) to generate stable ionizable hydrazones. Using this method, we were able to reliably quantify BA, 4HBA, 4-imidazole carboxaldehyde (4IA), and I3A in murine and human samples. We showed that gut microbes have the capacity to produce selected ArAs both *in vivo* and *in vitro* and that individuals with Crohn's disease (CD), but not those with ulcerative colitis (UC), have lower fecal levels of I3A and 4HBA relative to non-IBD controls.

## Materials and methods

### Research subjects

All subjects gave written informed consent, and study protocols were approved by the Cleveland Clinic Institutional Review Board (IRB# 10-544 and 19-1035).

Human fecal samples from individuals on/off antibiotics (Abx) were obtained from the CARNIVAL study collected in the pilot phase under an approved protocol registered under ClinicalTrials.gov Identifier: NCT01731236 (version Submitted Date: November 16, 2012). Healthy volunteers (*n* = 6) were subjected to a cocktail of oral poorly absorbed Abx for 7 d (ciprofloxacin, metronidazole, and vancomycin or ciprofloxacin, metronidazole, vancomycin, and neomycin) as previously described.^25^ Volunteers were not pregnant and did not have chronic illness (including known history of heart failure, renal failure, pulmonary disease, gastrointestinal disorders, or hematologic diseases). Feces were collected at the baseline and following the 7-d Abx-cocktail treatment.

Additionally, biobank fecal samples from subjects with IBD (CD + UC; *n* = 30 and non-IBD controls; *n* = 16) included patients attending a routine visit, who fulfill the following selection criteria: diagnosis of CD or UC based on accepted guideline criteria,^26^^,^^27^ age 18–70 y, and any disease location except isolated upper gastrointestinal CD. Exclusion criteria included antibiotic use within three months prior to the study, status postcolectomy, colostomy or ileostomy, chronic liver, renal, lung or metabolic disorders, active malignancy, and gastrointestinal infections. The stool was collected by the participating study subjects into a sterile container and kept at 4 °C until processing. Stool was homogenized using a sterile spatula and then aliquoted and stored at −80 °C.

During laboratory analyses of all human samples, personnel were blinded to all but sample identifier (bar code). Upon completion of lab analyses the database coordinator provided information on the group status.

### Animal studies

All animal studies were conducted under protocols approved by the Cleveland Clinic Institutional Animal Care and Use Committee (IACUC; protocol# 2750).

The impact of the gut microbiome on ArA levels was studied in 8–10-week-old C57BL/6J mice (The Jackson Laboratory, Bar Harbor, ME), comprising a total of 20 male and 19 female mice fed a standard chow diet (2918 Teklad Irradiated Global 18% Protein Rodent Diet, Inotiv, IN) in conventional housing with a 12 h/12 h light/dark cycle in rodent cages housing five animals per cage. The mice were randomized into cages by personnel blinded to the study experimental design and then divided into two groups: the Abx group was treated with a cocktail of Abx previously shown to suppress gut microbiota^28^ by providing kanamycin (0.4 mg/mL), gentamicin (0.035 mg/mL), colistin (0.057 mg/mL), metronidazole (0.215 mg/mL), vancomycin (0.045 mg/mL), and erythromycin (0.01 mg/mL) in drinking water for four weeks, while the control mice were given regular drinking water. Food consumption and body weight were measured weekly. Food intake was assessed by normalizing the food consumed per day to the average body weight within a cage.

To verify if Abx treatment reduced the bacterial load, the fecal DNA was extracted from fecal samples using the DNeasy PowerSoil ProKit (Qiagen, Germantown, MD, Cat# 47016), following manufacturer's instructions. The total fecal DNA concentration was quantified using Nanodrop (Thermo Fisher Scientific, ND-2000) and normalized to the fecal weight. 16S DNA was quantified by quantitative PCR using the following primers: forward: 5′-CTCCTACGGGAGGCAGCAG-3′ and reverse: 5′-TTACCGCGGCTGCTGGCAC-3′. PCR reactions (20 µL) comprised 20 ng DNA (volumes were adjusted per sample based on the concentration) as template, 10 µL SYBRGreen Master Mix (Alkali Scientific, Fort Lauderdale, Fl, Cat# QS1020), 0.5 µL of each primer (4 µM final concentration), and nuclease-free water. PCR cycle conditions were 95 °C for 3 min, followed by 39 cycles of amplification (95 °C for 10 s, 60 °C for 30 s, 72 °C for 30 s).

### Reagents and chemicals

All organic solvents and mobile phases were prepared using LC/MS-grade chemicals and were purchased from Thermo Fisher Scientific (Waltham, MA) unless otherwise noted. BA, 4HBA, and I3A were purchased from Sigma-Aldrich (Burlington, MA); 4IA from Chem-Impex (Wood Dale, IL); 3MPH hydrochloride from Thermo Scientific Chemicals (Waltham, MA); and D_6_-benzaldehyde (D_6_-BA) with 99.5% isotopic purity from CDN Isotopes (Quebec, Canada). USP-grade Abx kanamycin, colistin, metronidazole, gentamicin, and vancomycin were purchased from GoldBio (St. Louis, MO), and erythromycin from Teknova (Hollister, CA).

### Standard and internal standard solutions

All ArA stocks and working standards were prepared in methanol and stored at −80 °C before use. ArA mixtures at nine different concentrations, ranging from 0 to 1000 nM, were prepared for the analysis of ArA in feces and bacterial cultures. An isotopically labeled internal standard (IS; D_6_-BA) master solution was prepared in methanol, and the IS working solution (10 µM) was prepared in acetonitrile and stored at −20 °C until use.

### Bacterial culture samples

All bacterial strains, media and growth parameters used in this study are shown in Supplementary Table S1. *Clostridium sp.*, *Eubacterium sp. 3_1_31*, *Faecalibacterium prausnitzii*, *Flavonifractor sp.*, *Coproccocus comes*, and *Dorea sp.* were grown in an anaerobic mixture (AM) media prepared from 50% Gifu anaerobe broth (HIMedia, Kennett Square, PA Cat# M1801; comprised of peptone 10 g/L, soya peptone 3 g/L, proteose peptone 10 g/L, digested serum 13.5 g/L, yeast extract 5 g/L, HM peptone B 2.2 g/L, HL extract 1.2 g/L, dextrose 3 g/L, KH_2_PO_4_ 2.5 g/L, NaCl 3 g/L, soluble starch 5 g/L, L-cysteine x HCl 0.3 g/L, and sodium thioglycollate 0.3 g/L) and 50% of Wilkins Chalgren Anaerobic Broth Base (HIMedia, Kennett Square, PA Cat# M863; tryptone 10 g/L, peptone 10 g/L, yeast extract 5 g/L, dextrose 1 g/L, NaCl 5 g/L, L-arginine 1 g/L, sodium pyruvate 1 g/L, hemin 0.005 g/L, and menadione 0.0005 g/L). *Enterococcus sp.* were grown in brain-heart infusion (BHI) broth (37 g/L; BD Difco, Franklin Lakes, NJ, Cat# B11059) composed of the following: BHI 6 g/L, peptic digest of animal tissue 6 g/L, pancreatic digest of gelatin 14.5 g/L, dextrose 3 g/L, NaCl 5 g/L, and Na_2_HPO_4_ 2.5 g/L. *Lactobacillus johnsonii* and *Limosilactobacillus* *reuteri* were grown in de Man, Rogosa and Sharpe (MRS) broth (55 g/L; BD Difco, Franklin Lakes, NJ, Cat# DF0881-17-5) composed of proteose peptone No. 3 10 g/L, beef extract 10 g/L, yeast extract 5 g/L, dextrose 20 g/L, polysorbate-80 1 g/L, ammonium citrate 2 g/L, sodium acetate 5 g/L, MgSO_4_ 0.1 g/L, MnSO_4_ 0.05 g/L and K_2_HPO_4_ 2 g/L. *Bacteroides thetaiotaomicron* strains were grown in tryptone-yeast extract-glucose (TYG) broth prepared as follows: Tryptone Peptone (10 g/L; Gibco, Cat. # 211921), Bacto Yeast Extract (5 g/L; BD, Cat# 212750), glucose (2 g/L; Sigma, Cat. # G8270), cysteine x HCl (0.5 g/L; Sigma, Cat# C1276), 1 M potassium phosphate buffer (pH 7.2, 100 mL/L) made using 1 M potassium phosphate monobasic (Sigma, Cat. # P0662), and 1 M potassium phosphate dibasic (Sigma, Cat# P3786), vitamin K3 (menadione) (1 mL/L of a 1 mg/mL solution; Sigma, Cat# M5625), TYG Salts (40 mL/L) made using MgSO_4_ × 7H_2_O (0.5 g/L; Sigma, Cat# M63-500), NaHCO_3_ (10 g/L; Sigma, Cat# S4019), NaCl (2 g/L; Sigma, Cat# S3014), FeSO_4_ (1 mL/L of a 0.4 mg/mL stock; Sigma, Cat# F8048), CaCl_2_ (1 mL/L of an 8 mg/mL stock; Sigma, Cat# C7902), hemin (0.5 mL/L of a 10 mg/mL solution; Sigma, Cat# 51280), and vitamin B12 (0.5 mL/L of a 0.01 mg/mL solution; Sigma, Cat# V2876).

All anaerobic cultures were grown at 37 °C in an anaerobic chamber (Coy Manufacturing, Grass Lake, MI) with an atmosphere of compressed gases (5% CO_2_, 5% H_2_, and 90% N_2_, with hydrogen set to ~2.5% in the growth chamber) and aerobic cultures were grown at 37 °C in VWR Air Jacketed CO_2_ Incubator with 5% CO_2_. Cell-free conditioned media were obtained from 5 ml cultures of bacteria that were inoculated from single colonies and grown until the stationary phase. Cultures were centrifuged to pellet bacteria (8000 × *g*, 4 °C, 5 min), and the supernatant was passed through a 0.22 µm filter (MilliporeSigma, Burlington, MA). The control media was processed using the same method.

### Sample preparation and derivatization

Fecal pellets were stored at −80 °C until use. Extraction buffer (100% methanol; 100 µL/10 mg of feces) was added to the fecal samples and vortexed for 5 min and then incubated at −20 °C for 40  min, followed by centrifugation (14,000 × *g*, 20  min, 4 °C). The supernatant (20 µL) was mixed with an ice-cold methanolic solution of D_6_-BA (IS, 10 µL) and 3MPH (1 mg/mL in acetonitrile, 80 µL), vortexed, and centrifuged (14,000 × *g*, 20 min, 4 °C). The supernatant (75 µL) was transferred to glass HPLC vials with microinserts and incubated in the dark for four hours.

### LC-MS/MS conditions

LC‒MS/MS analysis was performed on a Shimadzu chromatographic system in tandem with Shimadzu 8050 triple quadrupole mass spectrometers (Shimadzu Scientific Instruments, Inc., Columbia, MD, USA). A Synergi™ Fusion-RP column (100 × 2.0 mm; 2.5 μm) (Cat# 00D-4423-AN, Phenomenex, Torrance, CA) was used for analytes separation. The mobile phase was made of 0.1% formic acid in water (Solvent A) and 0.1% formic acid in acetonitrile (Solvent B) under the following gradient: 0.0 min (0% B), 0.0–8.0 min (0% to 100% B), 8.0–10.0 min (100% B), 10.0–11.0 min (100% to 0% B), and 11.0–12.0 min (0% B) at a flow rate of 0.4 mL/min with a 3 µL injection volume. The column was maintained at 40 °C. Electrospray ionization in positive ion mode with multiple reaction monitoring (MRM) was used for analytes detection (see Results Table 1 for conditions), and the screen was set between 2.3 and 8.0 min. For data analysis, LabSolutions software 5.123 (Shimadzu) was used.

**Table 1.** UHPLC retention time (R.T.) and mass spectrometry MRM conditions for 3-MPH derivatives of ArA.

ArA	Abb.	R.T. (min)	Parent (*m/z*)	Fragment 1^*^ (*m/z*)	Fragment 2^#^ (*m/z*)	Q_1_ (V)	CE (eV)	Q_3_ (V)	IS
Benzaldehyde	BA	5.40	226.8	95.1	122.2	−11	−13	−11	D_6_-BA
4-Hydroxybenzaldehyde	4HBA	4.60	242.8	95.1	122.1	−12	−28	−15	D_6_-BA
4-Imidazolecarboxaldehyde	4IA	2.80	216.8	69.2	149.2	−11	−13	−27	D_6_-BA
Indole-3-carboxaldehyde	I3A	5.10	265.9	249.1	122.1	−14	−13	−11	D_6_-BA
D_6_-Benzaldehyde (IS)	D_6_-BA	5.40	231.3	95.1	190.2	−15	−28	−16	N/A

The table represents the results of mass spectrometry optimizing the conditions for the adducts between 3MPH and four ArAs, the retention time and the internal standard used for quantification. 3MPH = 3-methoxyphenylhydrazine; ArA = aromatic carboxaldehyde; Abb. = abbreviation; R.T. = retention time; IS = internal standard; MRM = multiple reaction monitoring; Q = quadrupole; CE = collision energy; *m/z* = mass‒to-charge ratio; N/A = not applicable; *Quantifying ions; ^#^Confirming ions.

### Linearity, limit of detection, limit of quantification, matrix effect, freeze–thaw and autosampler stability, and recovery

The spike-and-recovery approach was used to generate calibration curves. Calibration curves were built by fitting each analyte concentration to the analyte/IS peak area ratios. The limit of detection (LOD) was defined as the lowest concentration of analyte in a sample matrix that generated a signal-to-noise ratio of ≥3. The limit of quantification (LOQ) was defined as the lowest concentration of analyte in a sample matrix that generated a signal-to-noise ratio of ≥10. The matrix effect (ME) was determined by preparing three calibration curves in fecal extract pools and comparing their slopes to the average slope of three calibration curves prepared in methanol. The percentage matrix effect (%ME) was calculated using the following formula: %ME = (average slope of fecal pool calibration curve/average slope of methanol calibration curve) × 100. Freeze–thaw stability was measured in freshly homogenized fecal samples and aliquots that underwent the indicated number of freeze/thaw cycles. Autosampler stability was determined by storing derivatized quality control (QC) samples in an autosampler overnight operating at 4 °C and comparing the results to the nominal values of QC samples. Percent stability (%ST) was calculated using the following formula %ST = (measured average concentration after overnight storing in an autosampler/measured average concentration immediately after finished derivatization) × 100. Recovery was calculated by comparing the level of spiked samples in two different fecal pools before and after extraction and it was calculated as %Recovery = (amount recovered from samples spiked before extraction/amount recovered from samples spiked after extraction) × 100.

### Precision, accuracy, and quality control samples

Intraday and interday precisions were performed on three different QC samples. QCs were prepared by pooling multiple fecal pellets and homogenizing them in methanol followed by centrifugation and, if needed, spiking the pools with different amounts of synthetic ArAs. The supernatants were stored at −80 °C in O-ring cryovials prior to use. The precision of quantification was measured as the intraday and interday coefficients of variation. The intraday precision was determined by injecting six to eight analytical replicates of three QCs in a single day. The interday precision was determined by analyzing three levels of QC samples across eight runs on four separate days. Accuracy was determined by a standard addition method for the three QC levels in triplicate (accepted concentration) and compared with values from six analytical replicates of the three QCs analyzed using the methods calibration curve in a single day for intraday accuracy and for six different days for interday accuracy (experimental concentration). Accuracy was calculated as percent accuracy according to the following formula: percent accuracy = ((accepted concentration − experimental concentration)/accepted concentration) × 100. At least three different QC samples were included within each batch of LC-MS/MS samples (at the beginning, middle, and the end of a batch) to monitor assay performance.

### High-resolution mass spectrometry

Chromatography separation was performed by an Agilent 1290 Infinity II LC System. Reverse phase chromatography was performed by injecting 10 μL of each sample on the same column used for targeted analysis using the same chromatographic conditions. After separation, metabolites were detected by Daltonics timsTOF Pro 2 mass spectrometer (Bruker Daltonics, Bremen, Germany). The timsTOF Pro was calibrated according to the manufacturer's guidelines by injecting sodium formate at the beginning of each analysis as an internal calibrant. The analysis was performed in positive ion mode using a vacuum insulated probe heated electrospray ionization (VIP-HESI) ion source. The end plate offset was 500 V, and the capillary voltage was 4500 V. Nitrogen was used as dry gas at a flow rate of 8 L/min, and the nebulizer gas was set at 2.0 bar. The dry temperature was 230 °C, and the sheath gas temperature was 400 °C, using a sheath gas flow of 4 L/min. The scan range was 65–300 *m/z* at a scan speed of 12 Hz and resolution of 60,000. The collision energy was fixed at 5 eV. The acquired raw data were converted into mzML format with ProteoWizard-MSConvert (https://proteowizard.sourceforge.io/).

MSDIAL (https://systemsomicslab.github.io/compms/) was used to process the mzML files and for data visualization and collision-induced dissociation mass spectra comparisons.

### LC-MS/MS analysis of gut-microbial metabolites from aromatic amino acids

Selected gut-microbial metabolites were quantified by a previously published LC-MS/MS method^29^ with some modifications. Briefly, an ice cold methanolic solution of ISs (80 µL; D_5_-phenylacetic acid; D_9_-phenylpropionic acid; D_6_-4-hydroxyphenylacetic acid; D_7_-*p*-cresol sulfate D_4_-serotonin; D_5_-indole-3-acetic acid and D_2_-indole-3-propionic acid) was added to the fecal extracts (20 µL), followed by vortexing and centrifuging (21,000 × *g*; 4 °C for 15 min). The clear supernatant was then transferred to glass vials with microinserts. An XSelect HSS T3 column (3.0 × 100 mm; 3.5 µm) (Cat# 186004780, Waters, Ireland) was used for chromatographic separation. A gradient of solvent A (0.1% acetic acid in water) and B (0.1% acetic acid in acetonitrile) was used for chromatographic separation with a flow rate of 0.4 mL/min and a 1 µL injection volume. Electrospray ionization in positive and negative ion mode was performed under the following MRM conditions: *m/z* 177.2 → 160.1 for serotonin; *m/z* 181.2 → 164.1 for D_4_-serotonin; *m/z* 161.0 → 144.1 for tryptamine; *m/z* 206.2 → 118.1 for indole-3-lactic acid; *m/z* 190.0 → 130.1 for indole-3-propionic acid; *m/z* 191.8 → 130.1 for D_2_-indole-3-propionic acid; *m/z* 176.0 → 130.1 for indole-3 acetic acid and *m/z* 181.2 → 134.2 for D_5_-indole-3-acetic acid in positive ion mode and *m/z* 135.0 → 91.0 for phenylacetic acid; *m/z* 140.2 → 95.9 for D_5_-phenylacetic acid; *m/z* 149.0 → 105.1 for phenylpropionic acid; *m/z* 158.2 → 114.1 for D_9_-phenylpropionic acid; *m/z* 162.9 → 91.0 for phenylpyruvic acid; *m/z* 150.9 → 106.9 for 4-hydroxyphenylacetic acid; *m/z* 157.1 → 112.9 for D_6_-4-hydroxyphenylacetic acid; *m/z* 163.1 → 119.1 4-hydroxyphenylacrylic acid; *m/z* 165.1 → 121.0 for 4-hydroxyphenypropionic acid and 3-hydroxyphenylpropionic acid; *m/z* 136.9 → 93.1 for 4-hydroxybenzoic acid; *m/z* 187.0 → 106.9 for *p*-cresol sulfate and *m/z* 193.9 → 114.1 for D_7_-*p*-cresol sulfate in negative ion mode.

### 16S analysis

The microbial DNA was extracted from fecal samples using the DNeasy PowerSoil ProKit (Qiagen, Germantown, MD, Cat# 47016), following manufacturer's instructions. The DNA samples were subjected to 16S rRNA gene amplification and sequencing using methods previously described.^30^ Raw 16S amplicon sequence and metadata were demultiplexed using split_libraries_fastq.py script implemented in QIIME1.9.1.^31^ Demultiplexed fastq file was split into sample specific fastq files using split_sequence_file_on_sample_ids.py script from Qiime1.9.1.60 Individual fastq files without nonbiological nucleotides were processed using Divisive Amplicon Denoising Algorithm (DADA) pipeline.^32^

### Shotgun metagenomics sequencing and bioinformatics analysis

Total RNA was isolated from frozen fecal samples using a commercially available RNA isolation kit (NucleoSpin RNA plus, Takara Bio, San Jose, CA) following the manufacturer's protocol. The quality and concentration of the RNA samples were measured by NanoDrop (Thermo Scientific NanoDrop 1000, Waltham, MA, USA), and samples were stored at −80 °C until further analysis. Microbial community RNA sequencing was performed using previously published methods.^33^ Briefly, sequencing libraries were generated using Qiagen QiaSeq Fast Select Kit (Cat# 334376). The sequencing libraries were sequenced on illumina's Novaseq platform (NovaSeq × 1.5B flow cell, 2 × 150 bp).

The quality assessment of the metagenomic sequences was performed following established protocols. Initially, raw sequencing reads underwent quality filtering through the Trimmomatic pipeline to enhance data integrity.^34^ The reads related to host DNA were removed by aligning them against the reference mouse genome (GRCm39) using the BBMap tool (https://sourceforge.net/projects/bbmap/), which efficiently eliminates nontarget sequences. Subsequently, the filtered reads were utilized for taxonomic and functional characterization using Metaphlan4 and Humann3, respectively.^35^ For the differential abundance analysis, we employed the random forest methodology integrated within the DAtest package.^36^ This involved comparing various differential abundance detection methods based on metrics such as the false discovery rate (FDR), area under the curve (AUC), empirical power, and false positive rate (FPR). Our benchmarking analysis indicated that lefseq and metagenomeSeq were the preferred methods for conducting these abundance assessments. Throughout the analysis, we maintained a significant threshold of *P* < 0.05. When necessary, we adjusted the *P* values to account for multiple comparisons, applying the Benjamini and Hochberg approach to mitigate the FDR. The statistical significance of individual species abundance was evaluated using Tukey's honest significant difference (HSD) test. Additionally, species abundance was analyzed in relation to metadata variables using Maaslin2.^37^

### Analyses of ArAT and rpoB gene abundance

To quantify the abundance of genes encoding for aromatic aminotransferase (*ArAT*) and a housekeeping gene encoding for the beta-subunit of bacterial RNA polymerase (*rpoB*) in the shotgun metagenome, we retrieved nucleotide sequences from the NCBI Protein database using Entrez Direct.^38^ The command esearch -db protein -query | efetch -format fasta_cds_na was used to fetch coding sequences in FASTA format as the reference database. We then cleaned the raw metagenomic reads by trimming adapters, filtering low-quality bases (Phred < 20), and removing short reads (<50 bp) with Trimmomatic (v0.39)^34^ under default Illumina paired-end settings. Next, the cleaned reads were mapped to the reference sequences using Bowtie2 (v2.4.5)^39^ in end-to-end sensitive mode (--sensitive). We built an index with the bowtie2 index command and aligned reads via bowtie2 -x gene_index −1 forward_reads.fastq −2 reverse_reads.fastq -S output.sam. High-quality alignments (AS ≥ 100) were filtered using SAMtools (v1.15),^40^ and SAM files were converted to the sorted BAM format. Finally, we created a count matrix using featureCounts from Subread (v2.0.3).^41^

### Additional statistical analysis

A Shapiro‒Wilk test was performed to assess if data sets follow a normal distribution. For normally distributed data, an independent samples t-test was used to evaluate the differences for experiments with two groups, while ANOVA with Šídák's multiple comparisons test was applied for experiments with three or more groups. When data normality assumptions were violated, a Mann‒Whitney test was used for comparison of two groups, and a Kruskal‒Wallis test with Dunn's multiple comparisons was employed for comparison of three or more groups. The difference was considered statistically significant when the *P*-value was less than 0.05. All the statistical analyses were performed using GraphPad Prism version 10 for Windows (GraphPad Software, Boston, Massachusetts, USA).

## Results

### Quantitative analysis of aromatic carboxaldehydes

**Figure 1.**
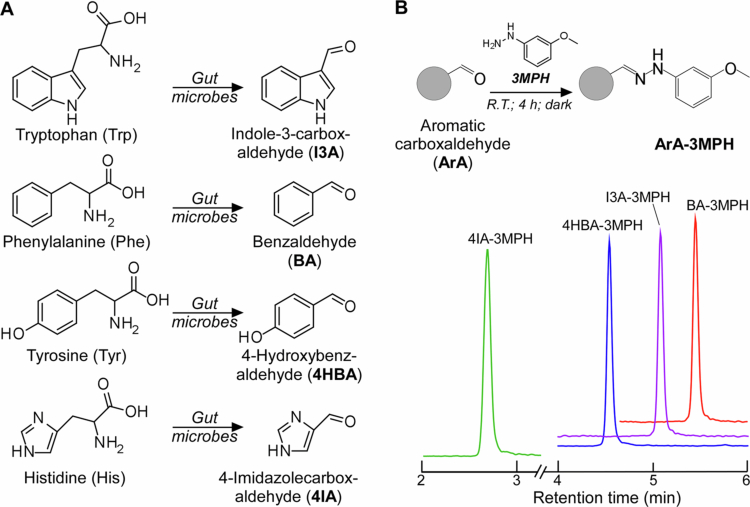
Aromatic carboxaldehyde analysis by LC-MS/MS. (A) Microbial production of aromatic carboxaldehydes (ArAs) from aromatic amino acids. (B) Derivatization of ArAs with 3-methoxyphenylhydrazine (3MPH), formation of hydrazone adducts (ArA-3MPH), and chromatographic separation of ArA-3MPH adducts (benzaldehyde (BA, red), 4-hydroxybenzaldehyde (4HBA, blue), 4-imidazolecarboxaldehyde (4IA, green) and indole-3-carboxaldehyde (I3A, purple)) on a SynergiTM Fusion-RP column (100 × 2.0 mm; 2.5 μm). The retention time and mass spectrometry parameters are given in Table 1.

**Figure 2.**
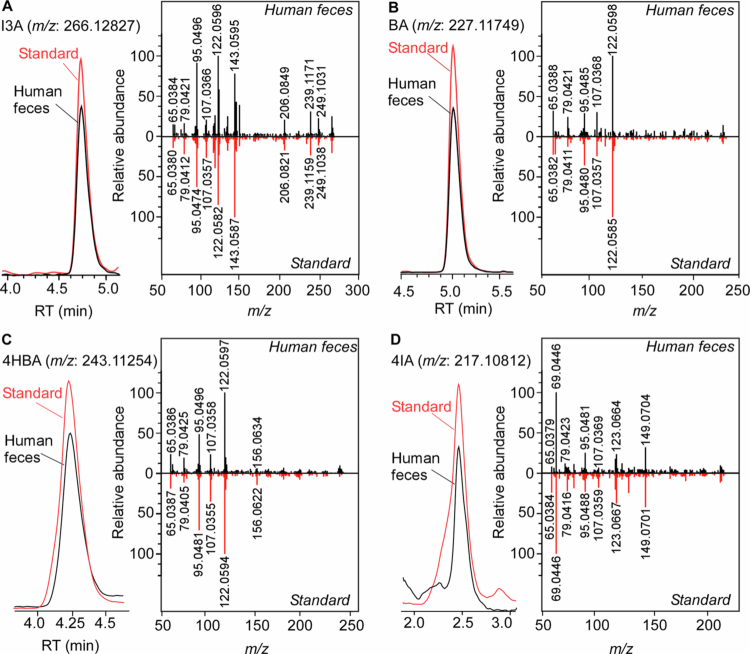
Comparison of high-resolution collision-induced dissociation mass spectra of pure synthetic standards for (A) indole-3-carboxaldehyde (I3A), (B) benzaldehyde (BA), (C) 4-hydroxybenzaldehyde (4HBA), and (D) 4-imidazolecarboxaldehye (4IA) and metabolites detected in human fecal extract after derivatization with 3-methoxyphenylhydrazine.

To study the role of gut microbes on ArAs production (Figure 1A), we first developed a stable isotope dilution quantitative LC-MS/MS method for their quantification. Due to poor ionizability of some ArAs (e.g. BA and 4HBA), ArAs were derivatized with 3MPH into stable hydrazones (Figure 1B). Optimization of electrospray mass spectrometry conditions was done by direct injection of each ArA-3MPH adduct into the mass spectrometer operating in positive ion mode. The final selected optimized LC-MS/MS conditions used for each ArA-3MPH adduct are given in Table 1. For the chromatographic separation of the ArAs, several reversed-phase chromatographic column matrices and conditions were tested. The best retention and separation from the derivatization reagent were achieved on SynergiTM Fusion-RP column (100 × 2.0 mm; 2.5 μm) (Figure 1B). We further confirmed that measured signals in human fecal extracts (Figure 2A–D) as well as mouse fecal extracts and bacterial cultures (Figure S1A–S1D and Table S2) match high-resolution mass spectra of the pure standards.

The method was further validated for intraday and interday precision and accuracy, matrix effect, freeze–thaw and autosampler stability as well as recovery. Intraday and interday precision for all four metabolites ranged from 4.4%–9.5% and 5.9%–12.6%, respectively, while intraday and interday accuracy for all four metabolites had respective ranges from 8.2%–18.3% and 5.7%–14.0% (Table S3). The LOD, defined as the lowest concentration of analyte in a sample matrix that generated a signal-to-noise ratio of ≥3, ranged from 7.5–15.9 nM. The LOQ, defined as the lowest concentration of analyte in a sample matrix that generated a signal-to-noise ratio of ≥10, ranged from 25.1 to 53.0 nM (Table S4). The matrix effect, determined by comparing calibration curve slopes in matrix vs methanol, was minimal with values ranging from 100.8% to 110.6%, and thus calibration curves for routine ArA analysis were prepared in methanol. The derivatized extracts were stable at 4 °C for up to 24 h postderivatization (Table S4). While the levels of I3A were not significantly reduced over three freeze–thaw cycles and 4HBA over two freeze–thaw cycles, the levels of 4IA and BA were significantly reduced after a single freeze–thaw cycle. The recovery ranged from 77.1% to 102.5% (Table S5). Based on the loss observed in our analysis, we do recommend a single-use aliquot strategy. All samples used in our study were aliquoted and immediately frozen prior to any analysis and none of them went through multiple freeze–thaw cycles.

### The impact of gut microbes on ArA production

**Figure 3.**
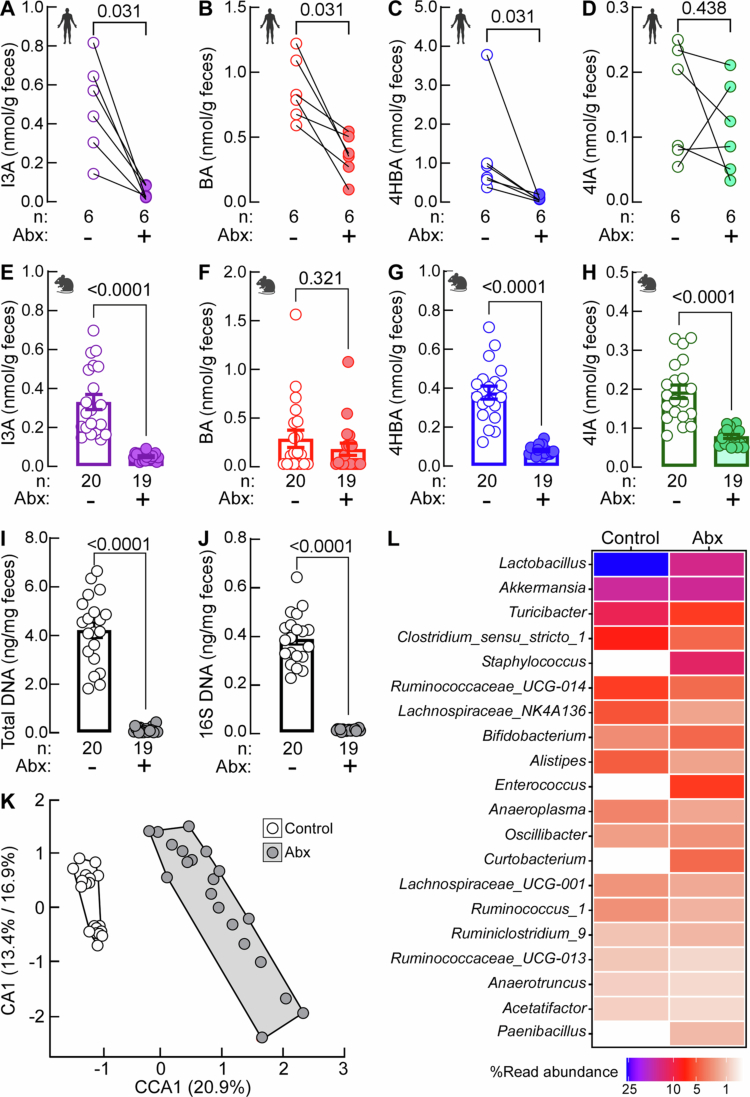
Gut-microbial presence affects fecal levels of aromatic carboxaldehydes. Concentrations of (A, E) indole-3-carboxaldehyde (I3A), (B, F) benzaldehyde (BA), (C, G) 4-hydroxybenzaldehyde (4HBA), and (D, H) 4-imidazolecarboxaldehyde (4IA) in (A-D) paired fecal samples from humans (*n* = 6) before and after a cocktail of antibiotics (Abx) and (E−H) mice on (*n* = 20) and off (*n* = 19) a cocktail of Abx. Fecal levels of (I) total and (J) 16S rDNA normalized to amount of feces in mice before and after a cocktail of Abx. (K) Canonical correlation analysis (CCA) of overall beta-diversity index between indicated groups and (L) heatmap of differentially abundant microbial species expressed as % read abundance among the indicated groups.

We next investigated the gut-microbial contribution to the fecal levels of individual ArA *in vivo*. First, we measured ArA levels in paired human fecal samples from healthy individuals before and after a 7-d course of a cocktail of oral poorly absorbed broad-spectrum Abx. Abx suppression of gut microbiota resulted in robust reduction of I3A, BA, and 4HBA (Figure 3A–C) suggesting a potential role of gut microbes in their production. 4IA was not uniformly affected by the treatment across individuals, most probably because its baseline levels were very low and on average 5–10 times lower than the other three ArAs but potentially reflecting inter-individual microbiome variability (Figure 3D).

Gut microbiota suppression studies were also performed in mice. Feces were collected from randomized male and female C57BL/6J mice following a four-week exposure to a cocktail of Abx and from control mice that were not exposed to Abx. Similar to the human studies, the levels of I3A and 4HBA were reduced by depletion of microbiota. In mice, fecal levels of 4IA were also reduced by microbial suppression, while the levels of BA were not affected by the treatment (Figure 3E–H). The effective suppression of the gut microbiota by our Abx regimen was supported by the significant reduction in total DNA and 16S rDNA gene at the experimental end point (Figure 3I and 3J). Furthermore, the provision of Abx resulted in altered microbiota composition as evidenced by separation of gut microbiota clusters (Figure 3K) as well as reduction in all main genera and overgrowth of *Staphylococcus*, *Enterococcus*, *Curtobacterium*, and *Paenibacillus* (Figure 3L).

Levels of selected ArAs were suppressed by the Abx treatment in both sexes (Figure S2A and S2B) and differences measured in control male and female mice were not significant (Figure S2C). These data suggest that ArA production is controlled by the gut microbiota in a sex-independent manner. We also noted the presence of residual amounts of ArAs post Abx. While the remaining ArAs could be produced by the bacteria resistant to Abx, they could also originate from the diet as we observed all four ArAs in the mouse chow (Figure S3A). However, the lower fecal ArA levels in mice on Abx were not due to decreased food intake consequent to Abx treatment, as no notable differences in body weight were observed between the Abx-treated and control groups at baseline or endpoint (after 4 weeks of Abx treatment) (Figure S3B). Furthermore, there were no differences in food uptake among both male and female mice on Abx compared to their respective controls (Figure S3C–D).

**Figure 4.**
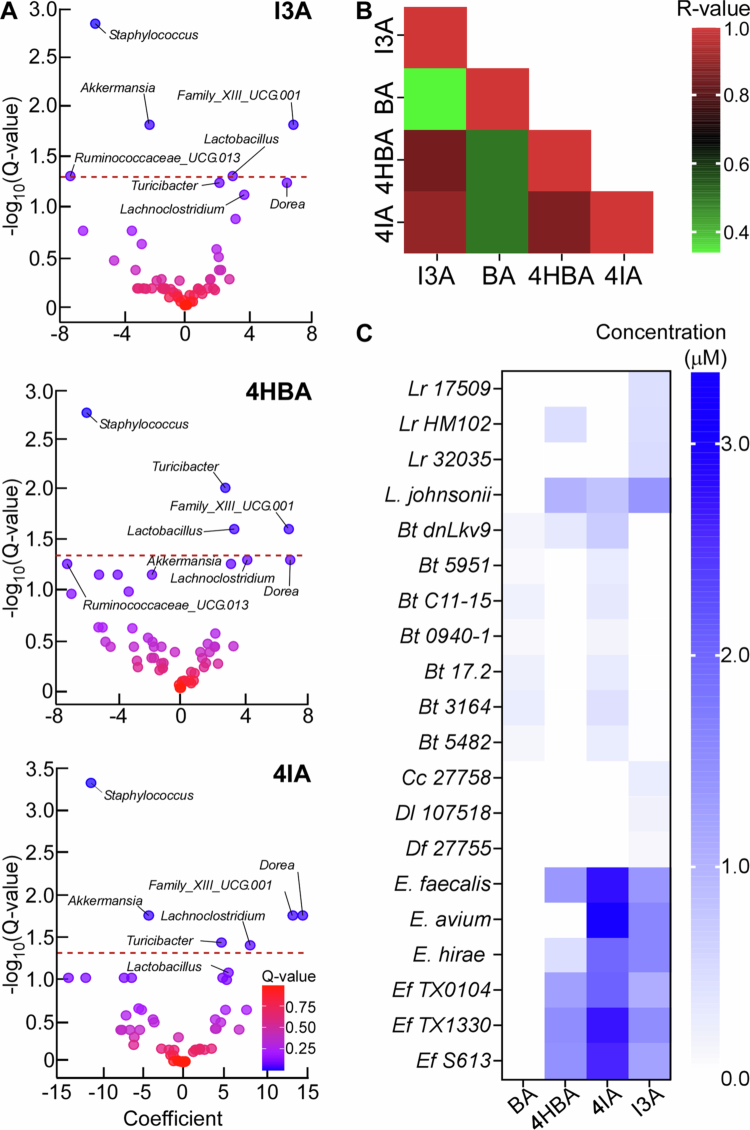
Multiple commensals have the capacity to produce aromatic carboxaldehydes. (A) Correlation coefficient vs Log_10_(Q-values) for indole-3-carboxaldehyde (I3A), 4-hydroxybenzaldehyde (4HBA), and 4-imidazolecarboxaldehyde (4IA) and identified microbial genera (red dotted line represents Q = 0.05). (B) Heat map showing Spearman correlation among levels of indicated pairs of ArAs in feces from mice not treated with Abx. (C) Level of ArAs produced by human commensals in culture. Each bar represents average values from three measurements of a single culture and represents one of minimum two independent experiments.

We also performed correlation analysis between metabolite levels and microbial genera in control groups and observed very similar correlation patterns for I3A, 4HBA, and 4IA. Those three ArAs were positively associated with genus *Family_XIII_UCG.001* and *Dorea* where the latter association reaches significance only for 4IA and negatively with genus *Staphylococcus*. 4HBA and 4IA were also positively associated with genus *Turicibacter*, I3A and 4HBA with *Lactobacillus*, while in 4IA the association did not reach statistical significance. Levels of 4IA were also positively associated with *Lachnoclostridium* (Figure 4A). Levels of BA were not associated with abundance of any genera (data not shown). Additionally, we observed a strong correlation among the measured levels of I3A, 4HBA, and 4IA (Figure 4B). This and similar association patterns with different microbial genera suggest that these ArAs share common metabolic pathways and/or producing microorganisms.

Next, we looked to identify specific members of the gut microbiota with the capacity to produce ArAs. To do so, we assessed the concentrations of ArAs in conditioned media (cell-free media conditioned by the growth of a given microorganism) from selected commensals that are prevalent in the human microbiota and/or previously known to produce ArAs (Methods, Table S1); data are summarized in Figure 4C. Our data revealed distinct patterns with respect to ArAs production. First, there was significant variation in the capacity of different species of bacteria to produce ArAs, highlighting how select commensals mediate this function within the microbiome. As previously established,^12^ we found that several isolates of *Limosilactobacillus reuteri* (*Lr*; previously *Lactobacillus reuteri*) produced I3A validating the utility of our method. In addition, some isolates of *Lr* also yielded 4HBA and 4IA. *Lactobacillus johnsonii* (*L. *johnsonii**) produced 4IA and 4HBA in addition to I3A. *Bacteroides thetaiotaomicron* (*Bt*) strains produced robust amounts of BA and 4IA, while 4HBA was prodcued only by the *dnLkv9* strain. In addition, this species also generated very small but detectable amounts of I3A. *Enterococcus* species produced the highest amounts of I3A and 4IA, in addition to 4HBA. We also note that in some experimental replicates, *E. faecalis* KB1 cultures, small amounts of BA were detected (not shown). Of note, all tested *Clostridia* strains failed to generate ArAs above the media background levels with *Coproccocus comes* (*Cc 27758*), *Dorea longicatena* (*Dl 107518*), and *Dorea *formicigenerans** (*Df 27755*) yielding small amounts of I3A (Figure 4C). Second, there was significant inter-strain variation both in respect to the capacity to produce a given ArA and in the amount produced. Thus, our data reveals distinct ArA production profiles when comparing strains, but also highlights extensive strain-level variation with respect to the type and quantity of production of individual ArAs.

### Aromatic carboxaldehydes and inflammatory bowel disease

To establish if identified ArAs are modulated in human disease, we quantified selected ArAs in feces from age and sex-matched individuals with IBD and compared them to the subjects without IBD (controls) (Table S6). IBD is a chronic inflammatory disorder of the gastrointestinal tract that is driven by pathogenic immune responses directed against microbiota, and in turn has impacts on microbiota composition. Thus, we reasoned that the abundance of ArAs may be affected during IBD, and furthermore, that the levels of select ArAs may correlate with disease.

**Figure 5.**
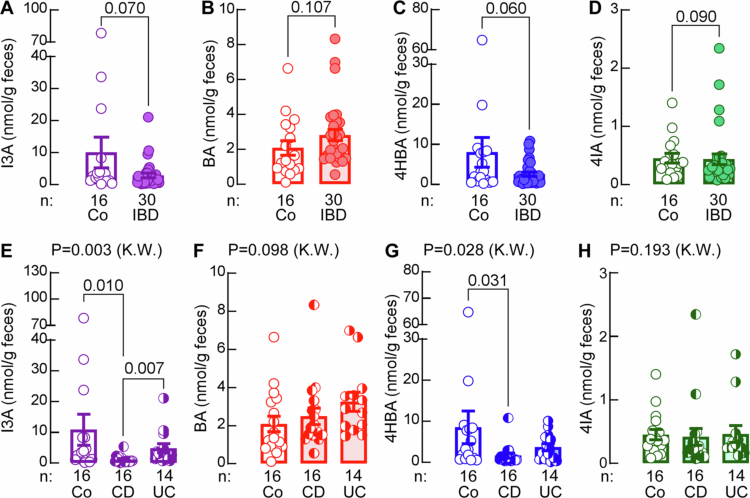
Individuals with Crohn's disease have lower fecal levels of indole-3-carboxaldehyde and 4-hydroxybenzaldehyde. Fecal levels of (A, E) indole-3-carboxaldehyde (I3A) (B, F) benzaldehyde (BA), (C, G) 4-hydroxybenzaldehyde (4HBA), and (D, H) 4-imidazolecarboxaldehyde (4IA) in non-IBD control subjects (Co, *n* = 16) and individuals with IBD (*n* = 30) or those with Crohn's disease (CD; *n* = 16) and ulcerative colitis (UC; *n* = 14). Mann-Whitney or Kruskal–Wallis with Dunn's multiple comparison were used for statistical analysis.

As depicted in Figure 5A–D, individuals with IBD had lower levels of I3A, 4HBA, and 4IA in their feces than control subjects, but the difference in levels was not statistically significant. However, after segregating individuals with IBD into those with CD vs UC, the two forms of IBD, individuals with CD, but not with UC, had significantly lower levels of I3A and 4HBA when compared to the controls. Moreover, levels of I3A were significantly lower in individuals with CD than those with UC (Figure 5E–H). Similar to what we observed in the mouse fecal samples, the levels of I3A and 4HBA also showed high positive correlation in human fecal samples (Figure S4A). Next, the levels of measured ArA values were compared to other indices and scores for IBD severity. We did not observe any significant correlations between fecal ArA levels and Crohn's Disease Patient-Reported Outcome 2 (CD-PRO2), Crohn's Disease Activity Index (CDAI) scores, Ulcerative Colitis Patient-Reported Outcome 2 (UC-PRO2), CDAI score, or calprotectin (Table S7).

In addition to ArAs, we measured other gut microbially produced metabolites that originate from the aromatic amino acids and observed no differences among the tested groups (Table S8) except for Tyr derived 4-hydroxyphenylacetic acid (4HPA) that was higher in feces from individuals with CD when compared to the non-IBD controls (Figure S4B).

**Figure 6.**
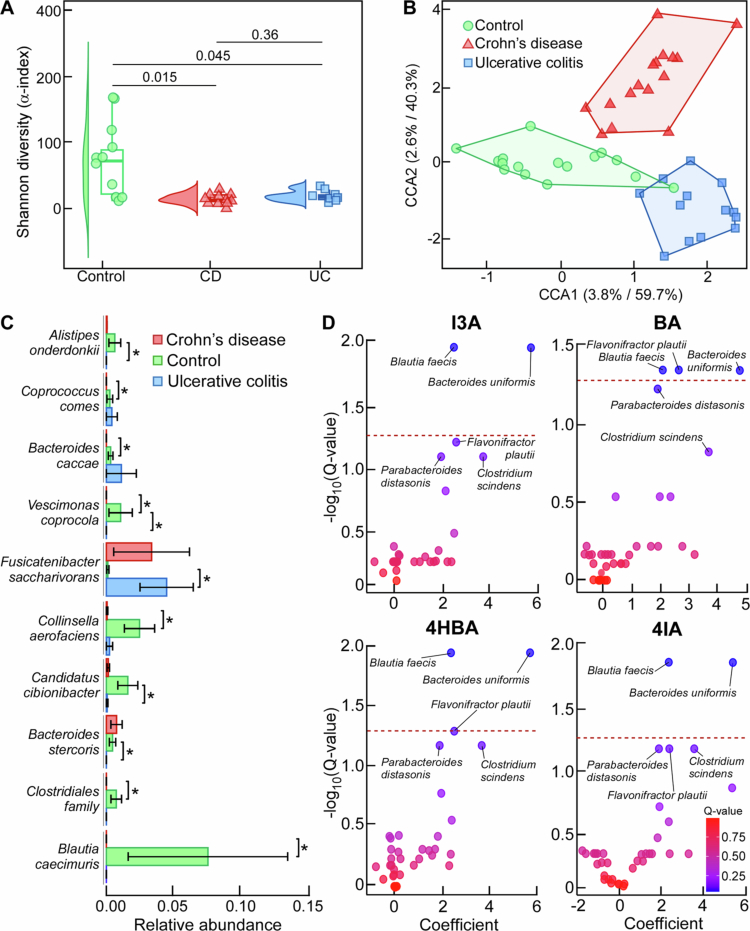
Inflammatory bowel disease affects gut-microbial structure. Metagenomic analysis of fecal samples from non-IBD control subjects (Co, *n* = 16), individuals with Crohn's disease (CD; *n* = 16) or ulcerative colitis (UC; *n* = 14). (A) Shannon diversity (alpha-index) and (B) canonical correlation analysis (CCA) of overall beta-diversity index among the indicated groups. (C) Differentially abundant microbial species expressed as relative abundance among the indicated groups. (D) Correlation coefficient vs Log_10_(Q-values) for indole-3-carboxaldehyde (I3A), benzaldehyde (BA), 4-hydroxybenzaldehyde (4HBA), and 4-imidazolecarboxaldehyde (4IA) and identified microbial species (red dotted line represents Q = 0.05).

Next, we performed metagenomics sequencing followed by bioinformatics analyses to determine the fecal microbiota composition of the individuals whose samples were used to quantify ArAs, allowing us to perform correlational analyses between the relative abundance of microbial taxa and levels of fecal ArAs. We observed lower Shannon diversity (alpha-index) in individuals with CD and UC vs the control group (Figure 6A). Canonical correlation analysis of the overall beta-diversity index shows separation of all three subgroups (Figure 6B). Differential abundance analysis revealed reduction in the relative abundance of *Alistipes onderdonkii*, *Candidatus cibionibacter*, and *Bacteroides stercoris* and increase of *Fusicatenibacter saccharivorans* in individuals with UC vs controls. According to the same analysis, *Coprococcus comes*, *Bacteroides caccae*, *Collinsella aerofaciens*, *Clostridiales family*, and *Blautia caecimuris* were lower in individuals with CD vs controls. *Vescimonas coprocola* was less abundant in both CD and UC when compared with controls (Figure 6C). All four ArAs were positively associated with abundance of *Bacteroides unformis* and *Blautia faecis*, while *Flavonifractor plautii* is also positively associated with levels of 4HBA and BA, while for I3A and 4IA it did not reach significance (Figure 6D). We also assessed *ArAT* expression in our clinical cohort, the only previously validated gene in the ArA metabolic pathway,^12^^,^^42^ and observed no significant differences among the tested groups despite our reported differences in ArA abundance.

## Discussion

Gut-microbially produced small molecules are emerging as important modulators of host health and disease susceptibility. IBD has been associated with gut-microbial dysbiosis characterized by reduced microbial diversity and disturbed balance between commensals and potential pathogens. Changes in the gut-microbial composition also leads to alterations in metabolites produced by the microbes and thus are likely to have a role in IBD pathogenesis.

The metabolic potential of gut microbes is still largely unexplored resulting in less than 1% of the annotated molecules in a typical untargeted metabolomic analysis to be classified as microbial.^43-45^ Additionally, poorly ionizable metabolites often are overlooked by classic untargeted LC-MS approaches. Therefore, new strategies in discovering and annotating gut-microbial metabolites are needed. In this study, we used 3MPH to ‘trap’ ArAs into ionizable hydrazones as a strategy to detect and quantify them by a robust, reliable, and reproducible stable-isotope-dilution LC-MS/MS method. The method should prove to be a valuable tool for further advancing the study of ArAs in context of host health and the exploration of links to gut microbiota-driven metabolism in the host. Furthermore, through the use of human and animal studies as well as bacterial cultures, we demonstrated that gut microbes have the capacity to produce ArAs beyond I3A. Specifically, we found that bacterial depletion by nonabsorbable antibiotics resulted in lower levels of I3A, BA and 4HBA in human feces and 4HBA, 4IA, and I3A in mouse feces. We also observed that multiple commensals have the capacity to produce ArAs in culture. Furthermore, we now note that several *Enterococci* have the capacity to produce an AhR agonist, I3A, at equivalent or higher levels of a previously known producer *L. reuteri* in addition to the other ArAs. Additionally, we observe positive correlation between mouse fecal I3A and 4HBA and the genus *Lactobacillus*. Although several tested strains did not produce ArAs, we cannot exclude the possibility that the ‘non-producers’ might yield them under different culturing conditions and further analyses are needed to establish their contribution to the ArA pool *in vivo*. Alternatively, select microbes may amplify the production of these molecules *in vivo* through yet unidentified microbe‒microbe interactions.

Previous studies showed that I3A has beneficial effects on intestinal barrier function by up-regulating the expression of tight-junction proteins, and through promotion of intestinal homeostasis.^15^ Additionally, both I3A and 4HBA ameliorated colitis in a dextran sodium sulfate (DSS)-induced colitis mouse model.^46^^,^^47^ Here, we show that gut microbes significantly contribute to the production of these ArAs and that individuals with IBD, specifically those with CD, have lower fecal levels of these two ArAs when compared to the non-IBD controls suggesting that modulating gut microbiome to increase production of these metabolites could be a strategy to mitigate some of the symptoms associated with these diseases.

Lower levels of I3A and 4HBA in individuals with CD, but not UC also suggest that the depletion of ArA is likely not simply attributable to the inflamed environment in the gastrointestinal tract of IBD patients but instead relates to specific facets of the pathogenesis of CD. The strong correlation between I3A and 4HBA levels suggests that those two ArAs share common metabolic pathways and/or producing microorganisms which are consistent with our *in vitro* data in which we show that many commensals can simultaneously produce both of those ArAs. Other gut microbially derived metabolites from the amino acid Trp have also shown beneficial effects on intestinal homeostasis, such as indole-3-propionic acid^48^ or indole-3-acetic acid.^12^^,^^49^ Here, we did not observe an association between the fecal levels of these indole acids, nor other Trp and Phe derived metabolites, with IBD suggesting that specific but not overall changes of bacterial metabolism of those aromatic acids are associated with this disease. The only other gut-microbial metabolite derived from aromatic amino acids measured in this study that was positively associated with CD was 4HPA, a phenolic acid derived from Tyr. Increases in 4HPA, a potential biosynthetic precursor of 4HBA, and reduction of 4HBA may suggest a disbalance in microbial metabolism of this amino acid in individuals with CD.

The loss of intestinal homeostasis or dysbiosis has been described in different intestinal disorders, including IBD. While a decrease in *F. saccharivorans* was previously observed in active UC, in contrast to the increase observed in patients with quiescent disease,^50^ here, we see an increase in individuals with UC when compared to non-IBD controls. Reduction in several other commensals in individuals with IBD when compared to non-IBD controls was also characteristic for our cohort. *A. onderdonkii*, *C. cibionibacter*, and *B. stercoris* were significantly lower in individuals with UC vs controls. According to the same analysis, *C. comes*, *B. caccae*, *C. aerofaciens*, *C. family*, and *B. caecimuris* were lower in individuals with CD vs controls.

Currently, endoscopy represents the best way of assessing the severity of IBD as well as the subtype of disease. However, its cost, inconvenience, risks, and accessibility make it less suitable for quick and frequent usage.^51^^,^^52^ The most widely available biomarkers in current clinical practice include serum and stool testing with C-reactive protein and fecal calprotectin and to a lesser extent lactoferrin. Given the emerging evidence supporting the relationship between disease progression and gut microbes, having biomarkers that reflect gut-microbial community output will be essential for establishing reliable signature molecules that will reflect changes in microbial composition and function in the context of IBD. Moreover, the capacity to distinguish between CD and UC through such tests will be of major utility for the clinical management of IBD. Robust, fast and reliable quantitative analytical methods for quantifying gut-microbial metabolites are essential for analyzing large clinical cohorts and identifying those biomarkers.

The present study has some limitations. In our method, we used a single IS. While we do observe high correlation between concentrations measured with D_6_-BA vs D_4_-HBA as IS across different sample types and concentration ranges (data not shown), additional validations will be required with compound-specific IS. We also note that this is an observational study with several unknown confounders that are not adequately adjusted for, particularly lack of dietary records and protein intake that can directly impact substrate availability for ArA production. Additionally, this is a relatively small cohort and additional ArA analysis in independent and validation cohorts is required.

## Supplementary Material

Kumar_ArA_Supplemental_materials_R2_01_23_2026 - clean.docx Kumar_ArA_Supplemental_materials_R2_01_23_2026 - clean.docx

## Data Availability

Data are available upon reasonable request. Metagenomes are deposited in NCBI under accession numbers SubmissionID: SUB14991076, BioProject ID: PRJNA1208041, and source data on zenodo.org doi: 10.5281/zenodo.14617594.

## Abbreviations

3MPH: 3-methoxyphenylhydrazine
4HBA: 4-hydroxybenzaldehyde
4IA: 4-imidazolecarboxaldehyde
Abx: antibiotic
AhR: aryl hydrocarbon receptor
ArA: aromatic carboxaldehyde
BA: benzaldehyde
CD: Crohn's disease
His: histidine
I3A: indole-3-carboxaldehyde
IBD: inflammatory bowel disease
IS: internal standard
LC-MS/MS: liquid chromatography-tandem mass spectrometry
LOD: limit of detection
LOQ: limit of quantification
ME: matrix effect
MRM: multiple reaction monitoring
Phe: phenylalanine
QC: quality control
SCFA: short chain fatty acid
Trp: tryptophan
Tyr: tyrosine
UC: ulcerative colitis
