# Supplementary material for: The role of gut microbes in production of aromatic carboxaldehydes
Source: Gut Microbes. 2026 Feb 21;18(1):2632979. doi: 10.1080/19490976.2026.2632979 (PMC12928648; doi:10.1080/19490976.2026.2632979)
Supplement: Kumar_ArA_Supplemental_materials_R2_01_23_2026 - clean.docx [file KGMI_A_2632979_SM1292.docx]

**Supplemental materials**

**The role of gut microbes in production of aromatic carboxaldehydes**

Manish Kumar^a,b#^, Rachel Son^a-c#^, Sarah M. Preston^a,b^, Robert W.P. Glowacki^a,b,†^, Kelley M. Carr^a,b^, Jiyeon Kim^a,b^, Jin Z. Ma^d^, Philip P. Ahern^a,b,e^, Jan Claesen^a,b,e^, Naseer Sangwan^a,b,e^, Florian Rieder^b,d-g^, and Ina Nemet^a,b,e*^

^a^Department of Cardiovascular & Metabolic Sciences, Cleveland Clinic Research, Cleveland, OH, USA

^b^Center for Microbiome & Human Health, Cleveland Clinic, Cleveland, OH, USA

^c^Department of Biology, Case Western Reserve University, Cleveland, OH, USA

^d^Department of Inflammation & Immunity, Cleveland Clinic Research, Cleveland, OH, USA

^e^Case Western Reserve University School of Medicine, Cleveland Clinic Lerner College of Medicine, Cleveland, OH, USA

^f^Department of Gastroenterology, Hepatology and Nutrition, Digestive Disease Institute, Cleveland Clinic, Cleveland, OH, USA

^g^Program for Global Translational Inflammatory Bowel Disease, Cleveland Clinic, Cleveland, OH, USA

^#^Authors contributed equally to this work.

^†^Current address: Hope College, Biology Department, Holland, MI

*Corresponding author: Ina Nemet, 9500 Euclid Ave, NC1-136, Cleveland Clinic, Cleveland, OH, USA 44195

Tel: +1 216 445 9578. Fax: +1 216 636 0392; Email: nemeti@ccf.org

**Table S1**. Bacterial culture, media and growth parameters.

| **Genus** | **Species** | **Abb** | **Strain** | **Growth media** | **Atmosphere** | **Growth time (h)** |
| --- | --- | --- | --- | --- | --- | --- |
| *Coproccocus* | *comes* | *Cc* | ATCC 27758 | AM | Anaerobic | 48 |
| *Dorea* | *longicatena* | *Dl* | DSMZ 107518 | AM | Anaerobic | 48 |
| *Dorea* | *formicigenerans* | *Df* | ATCC 27755 | AM | Anaerobic | 48 |
| *Clostridium* | *cadaveris* |  | HM-1039 | AM | Anaerobic | 48 |
| *Clostridium* | *scindens* |  | ATCC 35704 | AM | Anaerobic | 48 |
| *Clostridium* | *sporogenes* |  | ATCC 15579 | AM | Anaerobic | 24 |
| *Clostridium* | *clostridioforme* |  | DSMZ 26114 | AM | Anaerobic | 48 |
| *Enterococcus* | *faecalis* |  | KB1 | BHI | Aerobic | 16 |
| *Enterococcus* | *avium* |  | ATCC 49465 | BHI | Aerobic | 16 |
| *Enterococcus* | *hirae* |  | ATCC 10541 | BHI | Aerobic | 16 |
| *Enterococcus* | *faecium* | *Ef* | TX0104 | BHI | Aerobic | 16 |
| *Enterococcus* | *faecium* | *Ef* | TX1330 | BHI | Aerobic | 16 |
| *Enterococcus* | *faecium* | *Ef* | S613 | BHI | Aerobic | 16 |
| *Eubacterium* | *sp. 3_1_31* |  | HM-178 | AM | Anaerobic | 24 |
| *Faecalibacterium* | *prausnitzii* |  | KLE1255 HM-473 | AM | Anaerobic | 24 |
| *Flavonifractor* | *plautii* | *Fp* | DSMZ 26117 | AM | Anaerobic | 48 |
| *Flavonifractor* | *plautii* | *Fp* | ATCC 29863 | AM | Anaerobic | 48 |
| *Limosilactobacillus* | *reuteri* | *Lr* | DSM 17509 | MRS | Aerobic | 24 |
| *Limosilactobacillus* | *reuteri* | *Lr* | HM102 | MRS | Aerobic | 24 |
| *Limosilactobacillus* | *reuteri* | *Lr* | DSMZ 32035 | MRS | Aerobic | 24 |
| *Lactobacillus* | *johnsonii* | *Lj* | ATCC 33200 | MRS | Aerobic | 24 |
| *Bacteroides* | *thetaiotaomicron* | *Bt* | dnLkv9 | TYG | Anaerobic | 18 |
| *Bacteroides* | *thetaiotaomicron* | *Bt* | 5951 | TYG | Anaerobic | 18 |
| *Bacteroides* | *thetaiotaomicron* | *Bt* | C11-15 | TYG | Anaerobic | 18 |
| *Bacteroides* | *thetaiotaomicron* | *Bt* | 0940-1 | TYG | Anaerobic | 18 |
| *Bacteroides* | *thetaiotaomicron* | *Bt* | 17.2 | TYG | Anaerobic | 18 |
| *Bacteroides* | *thetaiotaomicron* | *Bt* | 3164 | TYG | Anaerobic | 18 |
| *Bacteroides* | *thetaiotaomicron* | *Bt* | 5482 | TYG | Anaerobic | 18 |

Abb: abbreviation; AM: anaerobe mix medium; BHI**:** brain heart infusion medium; MRS: De Man–Rogosa–Sharpe medium; TYG: tryptone yeast extract glucose medium.

**Table S2**. Comparison of experimental high-resolution collision induced dissociation mass spectral data of pure synthetic standards and metabolites detected in human and mouse fecal extract as well as bacterial conditioned media after derivatization with 3-methoxyphenylhydrazine.

| **Pure standards** | **Human feces** | | **Mouse feces** | | ***Enterococcus faecalis*** | | ***Lactobacillus jonsonii*** | |
| --- | --- | --- | --- | --- | --- | --- | --- | --- |
| ***m/z*** | ***m/z*** | **Error (ppm)** | ***m/z*** | **Error (ppm)** | ***m/z*** | **Error (ppm)** | ***m/z*** | **Error (ppm)** |
| **BA** | | | | | | | | |
| **227.11749** | **227.11771** | **-1.0** | **227.11730** | **0.8** | **227.11747** | **0.1** | **227.11745** | **0.2** |
| 122.0585 | 122.0598 | -10.7 | 122.0599 | -11.5 | 122.0594 | -7.4 | 122.0596 | -9.0 |
| 107.0357 | 107.0368 | -10.3 | 107.0366 | -8.4 | 107.0342 | 14.0 | 107.0359 | -1.9 |
| 95.0480 | 95.0485 | -5.3 | 95.0489 | -9.5 | 95.0490 | -10.5 | 95.0487 | -7.4 |
| 79.0411 | 79.0421 | -12.7 | 79.0414 | -3.8 | 79.0414 | -3.8 | 79.0414 | -3.8 |
| 65.0382 | 65.0388 | -9.2 | 65.0386 | -6.2 | 65.0384 | -3.1 | 65.0384 | -3.1 |
| **4HBA** | | | | | | | | |
| **243.11254** | **243.11480** | **-9.3** | **243.11192** | **2.6** | **243.11244** | **0.4** | **243.11277** | **-0.9** |
| 156.0622 | 156.0634 | -7.7 | 156.0645 | -14.7 | 156.0620 | 1.3 | 156.0603 | 12.2 |
| 122.0594 | 122.0597 | -2.5 | 122.0592 | 1.6 | 122.0594 | 0.0 | 122.0595 | -0.8 |
| 107.0355 | 107.0358 | -2.8 | 107.0359 | -3.7 | 107.0361 | -5.6 | 107.0368 | -12.1 |
| 95.0481 | 95.0496 | -15.8 | 95.0487 | -6.3 | 95.0487 | -6.3 | 95.0489 | -8.4 |
| 79.0405 | 79.0425 | -25.3 | 79.0414 | -11.4 | 79.0413 | -10.1 | 79.0413 | -10.1 |
| 65.0387 | 65.0386 | 1.5 | 65.0378 | 13.8 | 65.0390 | -4.6 | 65.0386 | 1.5 |
| **4IA** | | | | | | | | |
| **217.10812** | **217.10710** | **4.7** | **217.10715** | **4.5** | **217.10853** | **-1.9** | **217.10812** | **0.0** |
| 149.0701 | 149.0704 | -2.0 | 149.0697 | 2.7 | 149.0707 | -4.0 | 149.0707 | -4.0 |
| 123.0667 | 123.0664 | 2.4 | 123.0678 | -8.9 | 123.0680 | -10.6 | 123.0680 | -10.6 |
| 107.0359 | 107.0369 | -9.3 | 107.0361 | -1.9 | 107.0361 | -1.9 | 107.0361 | -1.9 |
| 95.0488 | 95.0481 | 7.4 | 95.0488 | 0.0 | 95.0485 | 3.2 | 95.0487 | 1.1 |
| 79.0416 | 79.0423 | -8.9 | 79.0407 | 11.4 | 79.0424 | -10.1 | 79.0417 | -1.3 |
| 69.0446 | 69.0446 | 0.0 | 69.0443 | 4.3 | 69.0447 | -1.4 | 69.0445 | 1.4 |
| 65.0384 | 65.0379 | 7.7 | 65.0383 | 1.5 | 65.0387 | -4.6 | 65.0381 | 4.6 |
| **I3A** | | | | | | | | |
| **266.12827** | **266.12901** | **-2.8** | **266.12781** | **1.7** | **266.12862** | **-1.3** | **266.12827** | **0.0** |
| 249.1038 | 249.1031 | 2.8 | 249.1042 | -1.6 | 249.1025 | 5.2 | 249.1025 | 5.2 |
| 239.1159 | 239.1171 | -5.0 | 239.1176 | -7.1 | 239.1178 | -7.9 | 239.1166 | -2.9 |
| 206.0821 | 206.0849 | -13.6 | 206.0838 | -8.2 | 206.0843 | -10.7 | 206.0817 | 1.9 |
| 143.0587 | 143.0595 | -5.6 | 143.0598 | -7.7 | 143.0603 | -11.2 | 143.0592 | -3.5 |
| 122.0582 | 122.0596 | -11.5 | 122.0597 | -12.3 | 122.0593 | -9.0 | 122.0593 | -9.0 |
| 107.0357 | 107.0366 | -8.4 | 107.0359 | -1.9 | 107.0359 | -1.9 | 107.0359 | -1.9 |
| 95.0474 | 95.0496 | -23.1 | 95.0493 | -20.0 | 95.0491 | -17.9 | 95.0491 | -17.9 |
| 79.0412 | 79.0421 | -11.4 | 79.0417 | -6.3 | 79.0416 | -5.1 | 79.0416 | -5.1 |
| 65.0380 | 65.0384 | -6.2 | 65.0385 | -7.7 | 65.0377 | 4.6 | 65.0388 | -12.3 |

BA: benzaldehyde; 4HBA: 4-hydroxybenzaldehyde; 4IA: 4-imidazolecarboxaldehyde; I3A: indole-3-carboxaldehyde; *m/z*: mass-to-charge ratio; Error (ppm): mass error in parts per million calculated as follows: [(standard *m/z* - sample *m/z*) / standard *m/z*] x 10^6^.

**Table S3**. Intraday and interday precision and accuracy.

| **ArA** | **QC level 1** | | | **QC level 2** | | | **QC level 3** | | |
| --- | --- | --- | --- | --- | --- | --- | --- | --- | --- |
|  | Mean (nM) | Precision  (%CV) | Accuracy  (%CV) | Mean (nM) | Precision  (%CV) | Accuracy  (%CV) | Mean (nM) | Precision  (%CV) | Accuracy  (%CV) |
| *Intraday* | | | | | | | | | |
| BA | 139.9 | 9.5 | 18.3 | 378.6 | 8.5 | 8.6 | 235.2 | 7.9 | 13.6 |
| 4HBA | 918.1 | 4.4 | 10.5 | 745.2 | 5.6 | 12.0 | 282.8 | 5.3 | 11.9 |
| 4IA | 79.1 | 4.9 | 14.0 | 135.9 | 6.4 | 10.7 | 204.3 | 5.5 | 16.7 |
| I3A | 439.2 | 5.2 | 11.8 | 241.5 | 6.1 | 15.3 | 188.3 | 5.6 | 13.8 |
| *Interday* | | | | | | | | | |
| BA | 139.6 | 11.1 | 13.6 | 379.2 | 10.7 | 8.3 | 234.5 | 10.9 | 11.5 |
| 4HBA | 918.0 | 6.6 | 13.2 | 745.6 | 6.5 | 13.9 | 282.8 | 5.9 | 9.3 |
| 4IA | 79.4 | 8.8 | 7.2 | 136.3 | 8.5 | 7.4 | 204.3 | 7.6 | 10.1 |
| I3A | 438.4 | 11.9 | 10.3 | 242.5 | 12.6 | 14.0 | 188.2 | 11.3 | 5.7 |

ArA: aromatic carboxaldehyde; BA: benzaldehyde; 4HBA: 4-hydroxybenzaldehyde; 4IA: 4-imidazolecarboxaldehyde; I3A: indole-3-carboxaldehyde; QC: quality control; CV: coefficient of variation; Intraday precision and accuracy were performed on three different QC samples. The intraday precision was determined by injecting eight analytical replicates of the three QCs in a single day and measuring the intraday CVs. Intraday accuracy was determined by a standard addition method for the three QC levels in triplicate (accepted concentration) and compared with values obtained from analytical replicates of the three QCs analyzed at the same day using the methods calibration curve (experimental concentration). Accuracy was calculated as percent accuracy according to the following formula: % accuracy = ((accepted concentration - experimental concentration)/accepted concentration) x 100; Interday precision and accuracy were performed on three different QC samples. The interday precision was determined by injecting eight analytical replicates of the three QCs in a single day over a span of four different days and measuring the interday CVs. Interday accuracy was determined by a standard addition method for the three QC levels in triplicate (accepted concentration) and compared with values obtained from analytical replicates of the three QCs analyzed over four days using the methods calibration curve (experimental concentration). Accuracy was calculated as percent accuracy according to the following formula: % accuracy = ((accepted concentration - experimental concentration)/accepted concentration) x 100.

**Table S4.** Limit of detection, Limit of quantification, Matrix effect and Autosampler stability.

| **ArA** | **LOD (nM)** | **LOQ (nM)** | **%ME** | **%ST** | | |
| --- | --- | --- | --- | --- | --- | --- |
|  |  |  |  | **QC level 1** | **QC level 2** | **QC level 3** |
| BA | 7.5 | 25.1 | 104.7±7.9 | 112.3±8.7 | 96.1±6.1 | 95.3±2.1 |
| 4HBA | 15.2 | 50.6 | 100.8±4.8 | 116.2±6.1 | 113.4±7.0 | 105.9±4.3 |
| 4IA | 15.9 | 53.0 | 110.6±1.4 | 112.9±12.5 | 107.8±13.5 | 103.0±8.6 |
| I3A | 8.1 | 27.1 | 107.0±10.9 | 101.2±14.2 | 106.3±16.9 | 98.3±14.9 |

ArA: aromatic carboxaldehyde; BA: benzaldehyde; 4HBA: 4-hydroxybenzaldehyde; 4IA: 4-imidazolecarboxaldehyde; I3A: indole-3-carboxaldehyde; LOD: limit of detection; LOQ: limit of quantification; ME: matrix effect; ST: stability; QC: quality control; The LOD was defined as the lowest concentration of analyte in sample matrix that generated a signal-to-noise ratio of ≥ 3. The LOQ was defined as the lowest concentration of analyte in sample matrix that generated a signal-to-noise ratio of ≥ 10. ME was determined by preparing calibration curves in fecal extract pools and comparing their slopes to the average slope in methanol. Percent matrix effect (%ME) was calculated using the following formula: %ME = (average slope of serum pool calibration curve / average slope of matrix matched calibration curve) x 100. Autosampler stability was determined by storing derivatized QC samples in an autosampler overnight operating at 4 °C and comparing the results to the nominal values of QC samples. Percent stability (%ST) was calculated using the following formula: %ST= (measured average concentration after overnight storing in autosampler/measured average concentration immediately after finished derivatization) x 100.

**Table S5**. Freeze-thaw stability and recovery.

| **ArA** | **Mean (nM)** | **%ST (P^1^)** | | | **%Recovery** |
| --- | --- | --- | --- | --- | --- |
|  |  | **Cycle 1** | **Cycle 2** | **Cycle 3** |  |
| *Fecal Slurry 1* | | | | | |
| BA | 165.3 | 79.5** | 78.4** | 83.4** | 77.1 |
| 4HBA | 314.2 | 95.0 | 98.2 | 94.4* | 102.5 |
| 4IA | 350.8 | 88.7** | 88.0** | 85.9** | 96.5 |
| I3A | 468.6 | 98.1 | 102.9 | 100.9 | 101.9 |
| *Fecal Slurry 2* | | | | | |
| BA | 136.9 | 81.6** | 83.5** | 88.3* | 77.4 |
| 4HBA | 515.5 | 91.8 | 97.9 | 92.7 | 94.3 |
| 4IA | 365.6 | 90.6* | 94.9 | 89.4* | 89.7 |
| I3A | 570.1 | 93.6 | 97.2 | 99.4 | 96.0 |

ArA: aromatic carboxaldehyde; BA: benzaldehyde; 4HBA: 4-hydroxybenzaldehyde; 4IA: 4-imidazolecarboxaldehyde; I3A: indole-3-carboxaldehyde; ST: stability; A fresh fecal sample was homogenized and divided into aliquots and froze at -80 °C. The aliquots were frozen/thawed 1-3 times. At each freeze/thaw cycle samples were taken for analysis. Percent stability (%ST) was calculated according to the following formula: %ST = (C_t_ / C_0_) x 100, where C_0_ is the concentration of ArAs in the fresh sample and C_t_ is the concentration after the indicated (t) freeze-thaw cycle. Recovery was tested by comparing the level of spiked samples in two different fecal pools before and after extraction and it was calculated as %Recovery = (amount recovered from samples spiked before extraction/amount recovered from samples spiked after extraction) x 100. ^1^P-values were calculated by Mann-Whitney test by comparing values after each freeze/thaw cycle with the first aliquot (*P<0.05; **P<0.001).

**Table S6.** Demographics and laboratory values of the participating subjects

| **Characteristics** | **Co (n=16)** | **CD (n=16)** | **UC (n=14)** | **P^1^**  **Co vs CD** | **P^1^**  **Co vs UC** |
| --- | --- | --- | --- | --- | --- |
| **Age (years)** | 59 (58-65) | 53 (40-63) | 39 (28-67) | 0.065 | 0.090 |
| **Sex (% male)** | 47 | 38 | 36 | 0.605 | 0.550 |
| **Calprotectin (µg/g)** | 1 (0-54) | 144 (60-217) | 69 (18-538) | <0.0001 | 0.013 |

Co: control subjects; CD: subjects with Crohn’s disease; UC: subjects with ulcerative colitis. Continuous data are presented as median (interquartile range), categorical variables are presented as %; ^1^P-values were calculated by Mann-Whitney test for continuous variables and Chi-square test for categorical variables.

**Table S7.** Spearman correlation coefficient between levels of ArAs and indices of IBD severity.

| **ArAs** | **CD-PRO2 score** | **UC-PRO2 score** | **CDAI** | **Calprotectin** |
| --- | --- | --- | --- | --- |
|  | ρ (p) | ρ (p) | ρ (p) | ρ (p) |
| **I3A** | -0.007 (0.985) | -0.041  (0.901) | 0.105  (0.749) | 0.047  (0.805) |
| **BA** | -0.106  (0.718) | -0.194  (0.541) | 0.357  (0.256) | 0.145  (0.444) |
| **4HBA** | -0.084  (0.776) | -0.131  (0.684) | -0.042  (0.904) | 0.199  (0.292) |
| **4IA** | -0.112  (0.701) | -0.041  (0.901) | 0.196  (0.543) | 0.164  (0.388) |

ArA: aromatic carboxaldehyde; BA: benzaldehyde; 4HBA: 4-hydroxybenzaldehyde; 4IA: 4-imidazolecarboxaldehyde; I3A: indole-3-carboxaldehyde; Spearman correlation coefficients were calculated between fecal levels of indicated ArAs and Crohn's Disease Patient-Reported Outcome 2 (CD-PRO2) score, Ulcerative Colitis Patient-Reported Outcome 2 (UC-PRO2) score, Crohn's Disease Activity Index (CDAI), and fecal levels of calprotectin. Correlation between ArAs and calprotectin included all individuals with IBD.

**Table S8**. Fecal levels of selected gut microbial metabolites derived from aromatic amino acids

| **Metabolite** | **Concentration (nmol/g feces)** | | | **P^1^** |
| --- | --- | --- | --- | --- |
|  | **Control (n=16)** | **CD (n=16)** | **UC (n=14)** |  |
| Serotonin | 1.35 (0.63-2.20) | 1.60 (1.09-2.84) | 1.13 (0.59-1.28) | 0.097 |
| Tryptamine | 1.84 (0.17-10.47) | 13.9 (0.26-59.1) | 1.14 (0.60-3.68) | 0.197 |
| Indole-3-lactic acid | 0.69 (0.31-0.90) | 0.31 (0.00-3.29) | 0.86 (0.41-1.04) | 0.656 |
| Indole-3-propionic acid | 3.21 (1.22-4.41) | 2.91 (1.06-4.19) | 4.16 (1.44-4.76) | 0.545 |
| Indole-3-acetic acid | 8.87 (4.90-17.39) | 5.27 (3.07-7.02) | 6.71 (3.22-8.34) | 0.220 |
| Phenylpyruvic acid | 4.56 (1.05-8.30) | 1.30 (0.76-2.38) | 1.26 (0.13-2.23) | 0.060 |
| Phenylacetic acid | 633 (230-952) | 482 (398-802) | 368 (299-575) | 0.246 |
| Phenylpropionic acid | 58.5 (18.0-107) | 15.19 (8.65-42.2) | 14.5 (0.00-96.5) | 0.169 |
| 4-OH-Phenylacetic acid | 12.80 (6.91-23.26) | 84.26 (15.44-278) | 12.5 (8.74-63.3) | **0.019** |
| 4-OH-Phenylacrylic acid | 0.65 (0.00-1.46) | 0.70 (0.25-1.07) | 0.34 (0.00-0.61) | 0.193 |
| 4-OH-Phenylpropionic acid | 0.00 (0.00-223) | 84.2 (0.00-157) | 17.30 (0.00-111) | 0.401 |
| 3-OH-Phenylpropionic acid | 64.78 (4.86-428) | 181 (54.8-364) | 152 (32.4-276) | 0.841 |
| 4-OH-Benzoic acid | 3.57 (1.78-7.20) | 4.21 (1.29-9.32) | 2.51 (1.67-6.50) | 0.837 |
| *p*-Cresol sulfate | 0.41 (0.21-1.83) | 0.23 (0.07-1.32) | 1.11 (0.27-3.37) | 0.399 |

CD: subjects with Crohn’s disease; UC: subjects with ulcerative colitis; OH: hydroxy; Values are expressed as median with interquartile ranges; ^1^P-values are calculated by Kruskal-Wallis test.

**
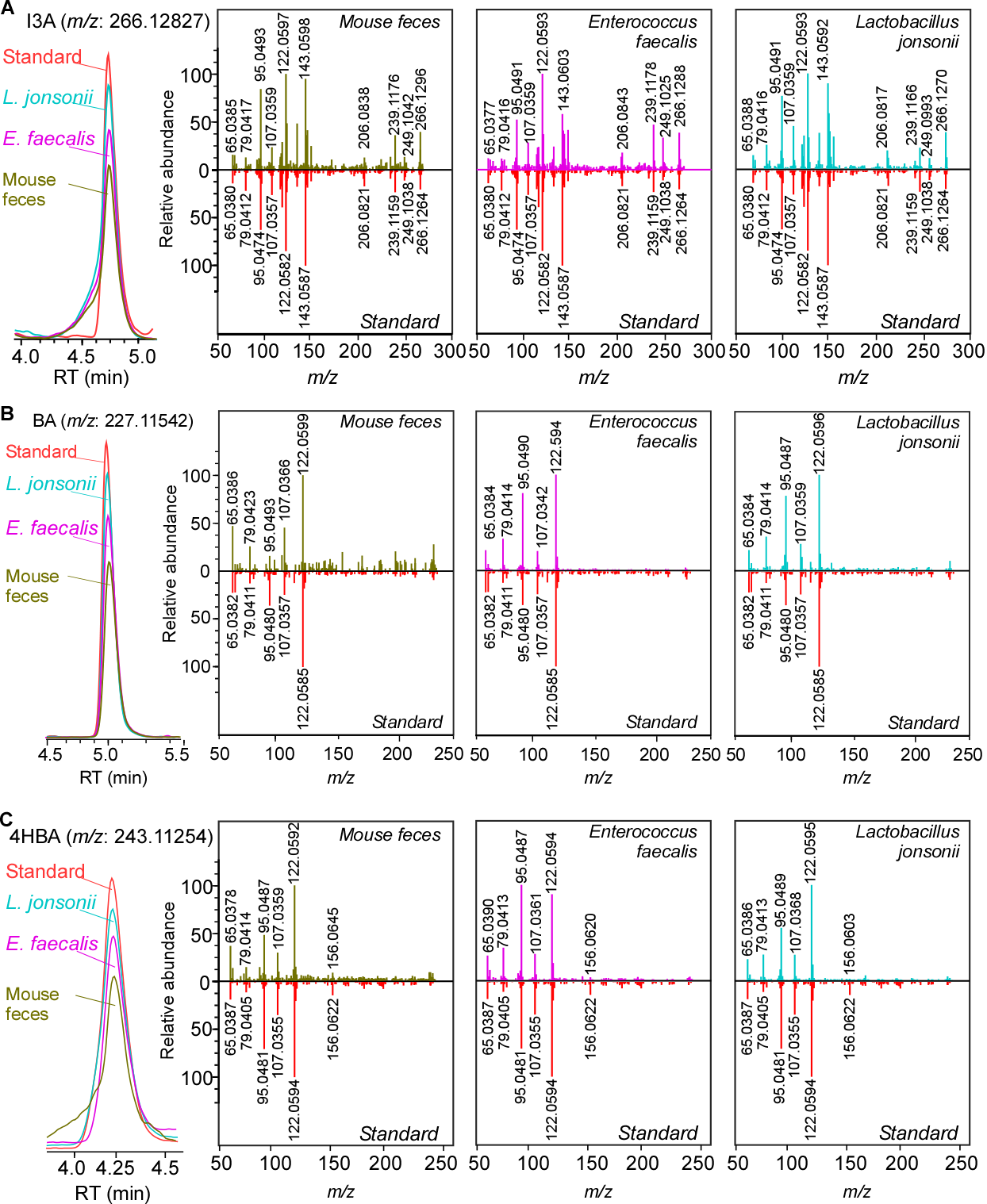
**

**
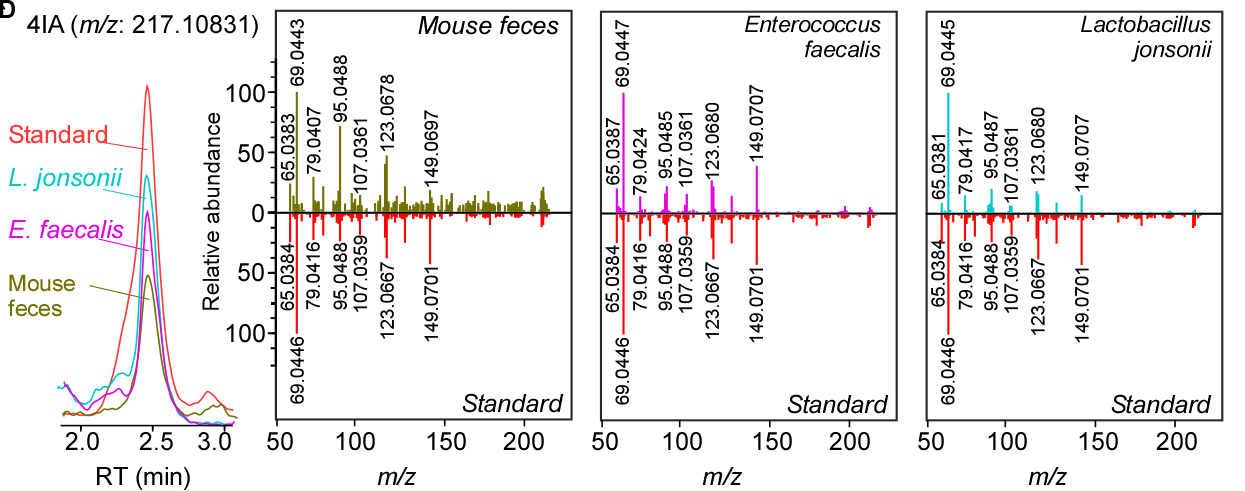
**

**Figure S1**. Comparison of high-resolution collision-induced dissociation mass spectra of pure synthetic standards for (**A**) indole-3-carboxaldehyde (I3A), (**B**) benzaldehyde (BA), (**C**) 4-hydroxybenzaldehyde (4HBA), and (**D**) 4-imidazolecarboxaldehye (4IA) and metabolites detected in mouse fecal extract and *E. faecalis* and *L. jonsonii* conditioned media after derivatization with 3-methoxyphenylhydrazine.


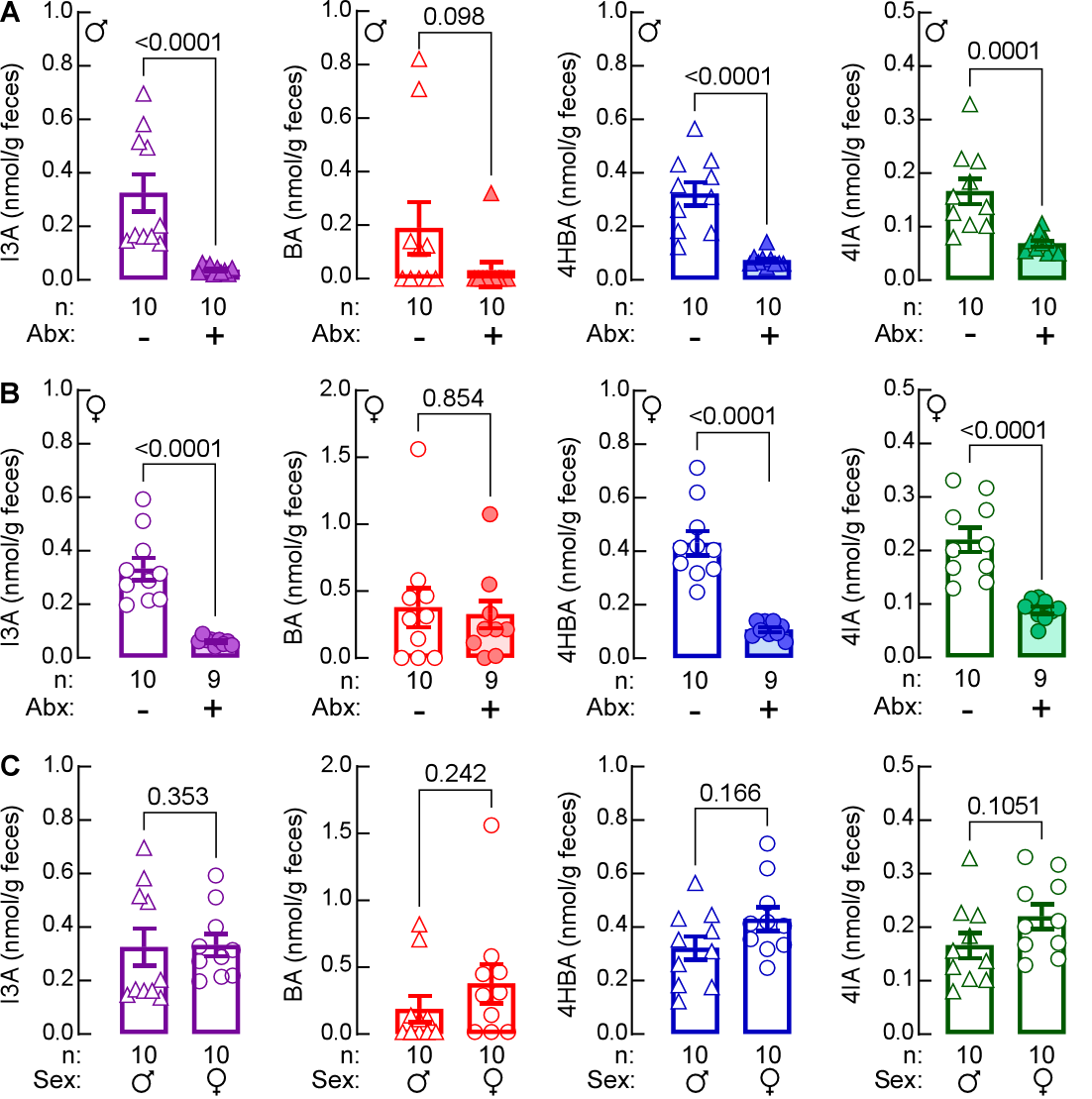


**Figure S2.** Fecal levels of indole-3-carboxaldehyde (I3A), benzaldehyde (BA), 4-hydroxybenzaldehyde (4HBA), and 4-imidazolecarboxaldehyde (4IA) in (**A**) male (n=10) and (**B**) female (n=9-10) mouse feces on and off a cocktail of antibiotics (Abx). (**C**) Comparison of ArA fecal levels in control male and control female mice; n=10 animals per group; Mann-Whitney test was used for statistical analysis.

**
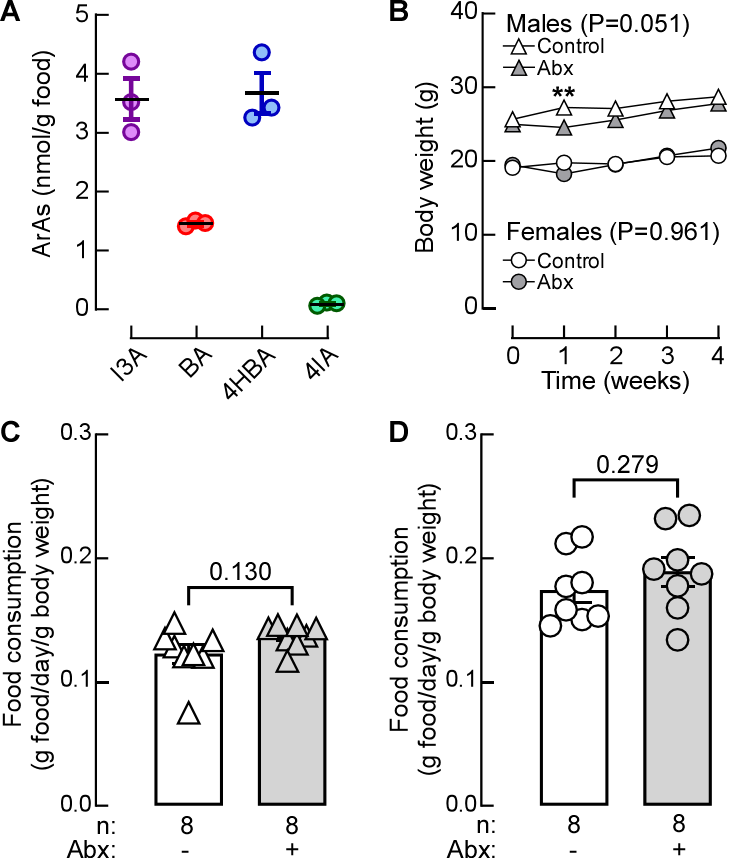
**

**Figure S3.** (**A**) ArA levels in mouse chow measured in 3 independent pellets; indole-3-carboxaldehyde (I3A), benzaldehyde (BA), 4-hydroxybenzaldehyde (4HBA), and 4-imidazolecarboxaldehyde (4IA); (**B**) Mouse body weight over the course of study in male (triangles; n=10) and female (circles; n=9-10) on (grey) and off (white) antibiotics (Abx). Average cage food consumption during the study in (**C**) male and (**D**) female mice on (grey) and off (white) Abx; n=2 cages per group over the 4-week time period. Two-Way ANOVA or Mann-Whitney test were used for statistical analysis.


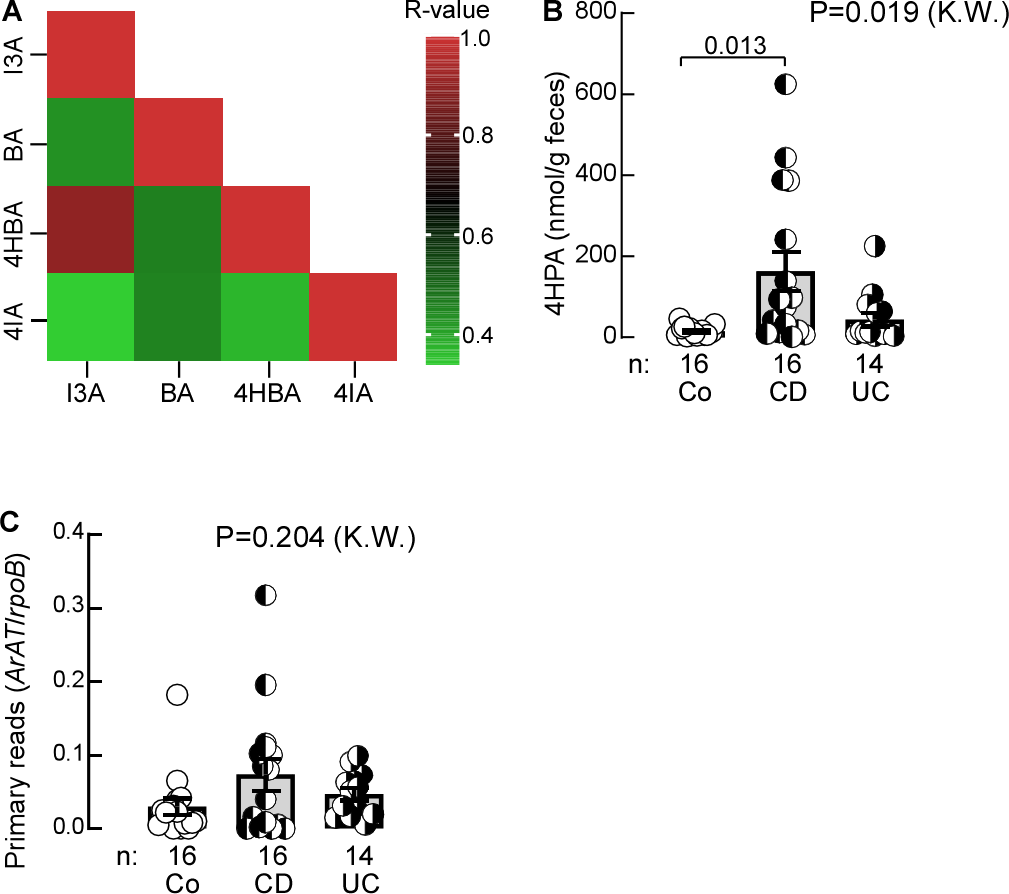


**Figure S4.** (**A**) Heat map showing Spearman correlation among indicated pairs of aromatic carboxaldehydes in human feces. (**B**) Fecal level of 4-hydroxyphenylacetic acid (4HPA) and (**C**) ratio between primary reads of genes encoding for aromatic aminotransferase (*ArAT*) and a housekeeping gene (beta-subunit of bacterial RNA polymerase (*rpoB*)) in control subjects (Co; n=16) and individuals with Crohn’s disease (CD; n=16) and ulcerative colitis (UC; n=14). Kruskal-Walis (K.W.) with Dunn’s multiple comparisons were used for statistical analysis.
